# Inflammatory biomarker outcomes associated with MDMA-assisted therapy: an open-label exploratory study

**DOI:** 10.3389/fnins.2026.1716817

**Published:** 2026-03-23

**Authors:** Jenna E. Kachmarik, Jennifer M. Loftis, Christopher S. Stauffer

**Affiliations:** 1 VA Portland Health Care System, Portland, OR, United States; 2 Department of Psychiatry, Oregon Health and Science University, Portland, OR, United States; 3 Department of Behavioral Neuroscience, Oregon Health and Science University, Portland, OR, United States

**Keywords:** CRP, IL-6, inflammation, inflammatory markers, MDMA, posttraumatic stress disorder, TNF-α, veterans

## Abstract

**Background:**

Posttraumatic stress disorder (PTSD) is associated with elevated inflammation and risk for chronic illness, yet few studies have examined inflammatory biomarker outcomes of PTSD interventions. Rapid PTSD symptom reduction has been observed following 3,4-methylenedioxymethamphetamine (MDMA)-assisted therapy, which leverages MDMA as a prosocial adjunct to psychotherapy. No studies have evaluated inflammatory biomarker outcomes of MDMA-assisted therapy. This exploratory pilot study examined within-person changes in inflammatory biomarkers during MDMA-assisted group therapy for Veterans with PTSD (www.clinicaltrials.gov, NCT05961527).

**Methods:**

Blood plasma samples were collected from 23 Veterans at baseline and end-of-intervention. Hedges’ g effect sizes were calculated for interleukin-6 (IL-6), tumor necrosis factor alpha (TNF-α), and C-reactive protein (CRP). PTSD severity was assessed with the Clinician-Administered PTSD Scale for DSM-5 (CAPS-5) at baseline and 30-day follow-up. Spearman’s rho correlations were calculated among biomarkers, PTSD symptoms, and change scores.

**Results:**

Small increases were observed in IL-6 (*g* = 0.24; 95% CI –0.25, 0.72) and CRP (*g* = 0.23; 95% CI –0.30, 0.74), and a small decrease in TNF-α (*g* = –0.24; 95% CI –0.69, 0.23). Baseline IL-6 and TNF-α were positively associated with baseline CAPS-5 scores (ρ = 0.45, 0.32). Higher baseline IL-6 weakly predicted symptom improvement (ρ = –0.25), and IL-6 change correlated with symptom change (ρ = 0.41). CRP showed weak negative associations with PTSD symptoms (ρ = –0.26).

**Conclusion:**

Findings suggest MDMA-assisted therapy may modulate inflammatory biomarkers and highlight biomarker–symptom relationships. Results are preliminary but may inform larger studies.

## 1 Introduction

Posttraumatic stress disorder (PTSD) is a serious mental health disorder affecting approximately 5% of adults in the United States, with estimates approaching 25% in some Veteran subpopulations (Dursa et al., 2014). Additionally, PTSD is associated with functional impairment (Jellestad et al., 2021) and poorer health outcomes—including a greater risk for developing several inflammatory health conditions (Klaric et al., 2017; O’Donovan et al., 2015).

Hypothalamic-pituitary-adrenal (HPA) axis dysregulation and its inflammatory sequelae are purported mechanisms linking PTSD to increased risk of physical health conditions (Lawrence and Scofield, 2024; Pace and Heim, 2011). The HPA axis regulates the stress response via a complex hormonal signaling cascade, facilitating physiological adaptation to environmental challenges. Glucocorticoids—including the “stress hormone” cortisol—are key mediators of the stress response (Smith and Vale, 2006). An adaptive stress response occurs transiently in response to acute stressors. Rising glucocorticoid concentrations bind glucocorticoid receptors in the hypothalamus and pituitary gland. Negative feedback inhibits further cortisol release, terminating the stress response (Smith and Vale, 2006). When stress is chronic, as with PTSD, the negative feedback loop is altered; glucocorticoid production and receptor sensitivity become dysregulated (Lawrence and Scofield, 2024; Pace and Heim, 2011). Consequently, downstream dysregulation of other physiological processes—including inflammation—may occur, as glucocorticoids suppress inflammatory activity (Dunn, 2007). Immune dysregulation, including alterations in a range of inflammatory biomarkers, has been observed across a variety of psychopathologies (Alessi and Bennett, 2020). Chronic stress is considered an etiological and/or exacerbating component of several psychiatric disorders (George et al., 2025), many of which demonstrate symptom overlap and exhibit similar patterns of inflammatory biomarker dysregulation (Alessi and Bennett, 2020). For example, in addition to PTSD, alterations in the pro-inflammatory biomarkers C-reactive protein (CRP), tumor necrosis factor alpha (TNF-α), and interleukin-6 (IL-6) have been observed in the setting of depressive and anxiety disorders (Camillo et al., 2025; Costello et al., 2019; Michopoulos et al., 2017). Thus, these markers are not specific to PTSD but may reflect inflammatory processes shared across multiple stress-related psychiatric disorders, as well as comorbid conditions (Madison et al., 2025).

PTSD appears to have a complex inflammatory profile; however, several meta-analyses demonstrate overall associations between PTSD and elevated levels of several circulating pro-inflammatory biomarkers, including CRP, TNF-α, and IL-6 (Passos et al., 2015; Yang and Jiang, 2020; Yang et al., 2025). Addressing PTSD symptoms may regulate inflammatory processes, as suggested by Gill et al. (2013), who observed similar CRP and IL-6 concentrations in women who had recovered from PTSD and healthy controls. These findings contrasted with significantly higher concentrations in women with a current PTSD diagnosis, though the data were cross-sectional, precluding conclusions of causality. The relationship between PTSD and inflammation is nuanced and bidirectional (Eraly et al., 2014; Katrinli et al., 2022; Sumner et al., 2020). Granted PTSD’s associations with inflammation and chronic illness, it is important to understand how interventions may influence these critical health factors.

Despite abundant research demonstrating a relationship between PTSD and inflammation (Li et al., 2025; Yang and Jiang, 2020; Yang et al., 2025), limited and conflicting empirical data exist concerning how PTSD interventions influence inflammation (Sagarwala and Nasrallah, 2019; Sumner et al., 2020). Few studies, using varied methodologies, have examined inflammatory biomarker outcomes associated with selective serotonin reuptake inhibitor (SSRI) medications, yielding mixed findings (Sagarwala and Nasrallah, 2019). Methodological heterogeneity also exists across a limited number of psychotherapeutic intervention studies in terms of inflammatory biomarker type and timing, psychotherapeutic intervention, psychological measures, and study design. Some have examined the predictive value of baseline inflammation in relation to PTSD symptom change (Maples-Keller et al., 2022; von Majewski et al., 2023). For example, Maples-Keller et al. (2022) reported that baseline CRP values did not predict PTSD symptom change in a sample of Veterans who completed a course of Prolonged Exposure. von Majewski et al. (2023) reported similar findings for CRP in a sample of adults who participated in trauma-focused cognitive behavioral therapy. Other studies have investigated the predictive effect of inflammatory reactivity to acute stress on PTSD treatment response (Renner et al., 2022; Rhein et al., 2021). Renner et al. (2022) reported that higher IL-6 levels in response to acute stress predicted worse therapy outcomes in an all-female sample of participants with PTSD; conversely, they reported that higher IL-10 levels in response to acute stress predicted better therapy outcomes. However, psychiatric outcome variables only included measures of general psychological burden and global impairment, with no direct PTSD symptom measurement. Rhein et al. (2021) also reported poorer therapy outcomes in a predominately female sample of individuals with PTSD for those with greater IL-6 reactivity in response to acute stress. Still other studies have evaluated inflammatory biomarker changes over the course of PTSD interventions (Himmerich et al., 2016; Toft et al., 2018; von Majewski et al., 2023; Zaccari et al., 2023). A study conducted by Himmerich et al. (2016) demonstrated increased TNF-α concentrations alongside decreased PTSD symptoms following inpatient and outpatient interventions for PTSD in an all-male sample of German soldiers with combat-related trauma; however, the concurrent changes were not correlated. Similarly, Toft et al. (2018) reported increases in TNF-α, as well as the pro-inflammatory biomarkers interleukin-1β (IL-1β) and monocyte chemoattractant protein-1, over the course of treatment in patients with PTSD compared to those without PTSD. The reported changes occurred alongside reductions in global mental health symptoms and general psychological distress; PTSD symptoms were not directly assessed. In their 2023 study, von Majewski reported finding no significant changes in CRP levels alongside improvements in PTSD symptom severity. Outcomes from Zaccari et al. (2023) demonstrated increased CRP levels 3 months after both Cognitive Processing Therapy (CPT) and a yoga-based PTSD intervention, both of which yielded similar PTSD symptom reductions. A greater CRP increase was observed in those receiving the yoga-based intervention. Significant increases in IL-6, IL-10, and the ratio of IL-10 to CRP were observed in the yoga-based intervention relative to the group receiving CPT. The study did not directly compare changes in inflammatory biomarkers to changes in PTSD symptoms. Given the variation in study design across extant literature, more research is needed to broaden our understanding of the impact of PTSD interventions on inflammation.

A promising investigational intervention for PTSD, 3,4-methylenedioxymethamphetamine (MDMA)-assisted therapy utilizes MDMA as a prosocial and supportive psychotherapy adjunct to strengthen therapeutic alliance and augment traumatic memory processing (Illingworth et al., 2021). Two phase 3 trials found that 67–71% of participants lost their PTSD diagnosis after MDMA-assisted therapy, demonstrating rapid and robust effects (Mitchell et al., 2021, 2023). Large effect sizes for MDMA-assisted therapy for PTSD, as compared to psychotherapy plus placebo, have been observed across multiple clinical trials (Illingworth et al., 2021; Mitchell et al., 2021, 2023). Findings have inspired research into potential mechanisms of efficacy—including translational research examining MDMA’s impacts on fear and traumatic memory processing, as well as its modulatory effects on neurotransmitter, neuroendocrine, and immune function (Feduccia and Mithoefer, 2018; Lewis and Byrne, 2023; Lewis et al., 2023; Parekh et al., 2022).

MDMA exhibits acute and short-term sustained immunosuppressive activity in animal models, as well as in humans with recreational use (Boyle and Connor, 2010; Parekh et al., 2022). These findings have limited generalizability to MDMA-assisted therapy for PTSD, where MDMA is given only a few times within a broader psychotherapeutic framework. Given evidence of rapid symptom improvement in MDMA-assisted therapy clinical trials (Illingworth et al., 2021; Mitchell et al., 2021, 2023) and established links among PTSD, inflammation, and chronic inflammatory illness (Passos et al., 2015; Yang and Jiang, 2020; Yang et al., 2025), examining outcomes of the full therapeutic modality merits further investigation.

To our knowledge, no prior study has examined inflammatory biomarkers in the context of MDMA-assisted therapy for PTSD, whether in individual or group formats. The present biomarker analysis was embedded within a single-arm, within-subjects, pilot study of an MDMA-assisted group therapy intervention for PTSD. Biomarkers of interest included IL-6, TNF-α, and CRP; these were chosen according to prior evidence linking these markers to PTSD symptoms and their use in studies investigating inflammatory processes associated with PTSD (Dell’Oste et al., 2023; Yang et al., 2025), including treatment-based interventions (Himmerich et al., 2016; Maples-Keller et al., 2022; Renner et al., 2022; Rhein et al., 2021; Sagarwala and Nasrallah, 2019; Toft et al., 2018; von Majewski et al., 2023; Zaccari et al., 2023). We examined inflammatory biomarker changes across the intervention and explored their associations with PTSD symptoms pre- and post-treatment. As greater inflammation is associated with PTSD, and MDMA-assisted therapy has demonstrated efficacy in PTSD symptom reduction, we anticipated MDMA-assisted group therapy would have anti-inflammatory effects.

## 2 Methods

### 2.1 Study design

This study was embedded within a parent clinical trial—a single-arm, open-label, pilot study investigating the feasibility and safety of a 12- to 16-week MDMA-assisted group therapy protocol for PTSD (NCT05961527, Stauffer et al., 2025).^1^ This sub-study was approved by the Portland VA Institutional Review Board.

### 2.2 Participants

Participants were U.S. military Veterans (*n* = 23) recruited as part of the parent study. Eligibility criteria, further detailed in Stauffer et al., 2025, required participants to have a charted PTSD diagnosis persisting for at least 3 months at the time of study enrollment and a concurrent score of ≥ 33 on the past-30-day PTSD Checklist for DSM-5 (PCL-5) at the time of screening.

### 2.3 Procedures

#### 2.3.1 Intervention

**FIGURE 1:**
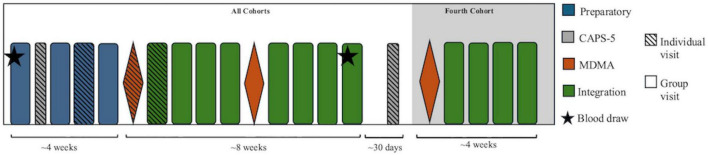
Timeline of MDMA-assisted group therapy (MDMA-AGT) sessions and assessments. The fourth cohort (shaded) included an additional treatment cycle. To standardize timing across all cohorts, variables of interest were measured after the same number of sessions as the first three cohorts.

The MDMA-assisted group therapy intervention was administered within the VA Portland Health Care System. Each of four cohorts consisted of five or six participants and a team of four co-facilitators. The first cohort consisted of women primarily with reported histories of military sexual trauma, the second and third cohorts were comprised of men with combat-related trauma histories, and the fourth cohort was made up of Veterans with a combination of military sexual and combat-related trauma histories. The study intervention (Figure 1) consisted of: (1) four preparatory sessions (three group sessions and one individual), (2) one individual MDMA session, (3) four integration sessions (one individual and three group sessions), (4) one group MDMA session, and (5) four group integration sessions. The intervention and the different session types are further detailed in the parent study protocol (Stauffer et al., 2025), which was later revised for the final cohort—based on participant feedback from the first three cohorts—to include an additional treatment cycle (i.e., a group MDMA session, followed by four additional group integration sessions). To ensure consistency in the number of study visits and timing of data collection across all cohorts, variables of interest were measured after the same number of therapy sessions as the first three cohorts; for all cohorts, this timepoint is referred to as the end-of-intervention.

#### 2.3.2 Blood collection

Non-fasting blood sample collection occurred at the VA Portland Healthcare System via a single venipuncture into K_2_EDTA blood collection tubes (BD Vacutainer Systems, Franklin Lakes, NJ, USA) at two timepoints. For all cohorts, baseline blood samples were collected within 4 h (average: 1 h and 18 min) before the start of the first therapy preparatory session. For the first three cohorts, follow-up blood samples were collected within 4 h (average: 1 h and 27 min) before the start of the final integration therapy session. As one participant did not attend the final scheduled group integration, their sample was collected prior to the second-to-last group integration. In the fourth cohort, follow-up corresponded to before the same integration session, even though this group had an additional treatment cycle pending approximately 1 month later. Blood samples were centrifuged at 3,200 rpm for 10 min. Plasma samples were aliquoted and stored at -20°C at the Portland VA Primary Care Laboratory and transferred within 2 weeks to a -80°C freezer for later immunoassay.

### 2.4 Measures

#### 2.4.1 Immunoassays

Measured inflammatory biomarkers included the pro-inflammatory biomarkers interleukin-6 (IL-6), tumor necrosis factor alpha (TNF-α), and C-reactive protein (CRP). Selection of inflammatory biomarkers used in analyses was based on previously established associations with PTSD symptoms (Passos et al., 2015; Yang and Jiang, 2020) and their use in prior studies examining inflammation–PTSD intervention relationships (Himmerich et al., 2016; Toft et al., 2018; von Majewski et al., 2023; Zaccari et al., 2023).

IL-6 and TNF-α plasma concentrations were individually assessed using high-sensitivity enzyme-linked immunosorbent assay (ELISA) kits; CRP was measured using a standard-sensitivity ELISA kit (R&D Systems, Inc., Minneapolis, MN, United States). For IL-6 and TNF-α, standard curves ranged from 0 to 10 pg/mL, both with a lower limit of quantification (LLOQ) of 0.156 pg/mL and upper limit of quantification (ULOQ) of 10 pg/mL. Reported sensitivities (lower limits of detection) for IL-6 and TNF-α were 0.09 and 0.049 pg/mL, respectively. The standard curve for CRP ranged from 0 to 50 ng/mL with a LLOQ of 0.78 ng/mL, a ULOQ of 50 ng/mL, and a reported sensitivity of 0.022 ng/mL.

Immunoassays were performed per manufacturer protocols at the Psychoneuroimmunology Laboratory (VA Portland Health Care System). For all analytes, standards and plasma samples were assayed in duplicate. Absorbance was measured using a Bio-Rad Model 680 Microplate Reader (Bio-Rad Laboratories, Inc., Hercules, CA, United States). Plates were read at optical densities (OD) of 450 and 540 nm. A wavelength correction was applied by subtracting the 540 nm absorbance values from the 450 nm values, in accordance with manufacturer recommendations. Interpolating the wavelength-corrected OD values against a seven-point standard curve yielded final concentration values. Standard curves were fit using a sigmoidal four-parameter logistic (4PL) model using Prism GraphPad (San Diego, CA, United States). Curve fit quality was evaluated using the coefficient of determination (*r*^2^), with all standard curves achieving *r*^2^ ≥ 0.96.

Following quality control procedures, plasma concentration data were retained for analysis if duplicate measurements were available for both timepoints and yielded a coefficient of variation (CV) ≤ 30%.

#### 2.4.2 PTSD

PTSD symptoms were assessed between the first and second preparation sessions (within 1 week of the initial blood draw; range: 1–6 days) and again 30 days post-intervention (approximately one to one-and-a-half months after the final blood draw; range: 25–47 days) (Figure 1) using the Clinician-Administered PTSD Scale for DSM-5 (CAPS-5) (Weathers et al., 2018), administered by independent, blinded raters. The CAPS-5 assesses PTSD diagnostic status and symptom severity using a structured interview format and demonstrates sound psychometric properties, including internal consistency (Cronbach’s alpha = 0.91), test-retest reliability (*r* = 0.82), convergent validity (*r* = 0.59) (Wojujutari et al., 2024), and discriminant validity (*r* = 0.20–0.54) (Weathers et al., 2018).

### 2.5 Statistical analysis

Participants who did not complete the full parent study protocol were excluded from analyses. For all inflammatory biomarker concentration values, natural log transformations were applied prior to analyses. Participants with measurements exceeding the reliable range of quantification at both timepoints were excluded from analyses, as no meaningful change could be approximated using these values. For three participants with CRP values above the ULOQ at one timepoint, data were retained by imputing the ULOQ for the out-of-range value. While it likely underestimates variance, this conservative approach retains usable within-subject comparisons and limits bias from complete-case exclusion (Herbers et al., 2021).

Statistical analyses were performed using IBM SPSS Statistics version 30. Effect sizes were calculated using the Hedges’ g statistic to correct for small sample size bias and interpreted using conventional thresholds (small ≈ 0.2, medium ≈ 0.5, large ≈ 0.8) (Lakens, 2013). This study emphasized effect size magnitude rather than null-hypothesis significance testing, given the exploratory nature and small sample size. To characterize estimation uncertainty, 95% confidence intervals are provided. Sensitivity analyses were conducted to assess: (1) CRP results when restricting analyses to participants with within-range concentrations at both timepoints and (2) outcomes when restricting to include samples with CV ≤ 20% at both timepoints.

Spearman’s rho correlations were calculated to evaluate relationships among inflammatory biomarker concentrations (baseline and end-of-intervention), CAPS-5 scores (baseline and 30 days post-intervention), and change scores for all variables, as this non-parametric measure is robust to outliers and appropriate for non-normally distributed data. Sensitivity analyses for correlations were conducted only for CRP, restricting to participants with within-range concentrations at both timepoints.

## 3 Results

Data from one participant were excluded from all analyses due to not completing the full parent study protocol, leaving a final sample size of 22. Demographic data and clinical characteristics are reported in Table 1. No findings were statistically significant in this small exploratory pilot study. Table 2 shows descriptive statistics and effect sizes for inflammatory biomarker concentration changes.

**TABLE 1:** Baseline demographics and clinical characteristics (*N* = 22).

Characteristic	Category	Value
Age; mean (SD)		42.4 (10.0)
Biological sex; *n* (%)	Male	14 (63.6%)
	Female	8 (36.4%)
Gender; *n* (%)	Male	11 (50%)
	Female	8 (36.4%)
	Other	3 (13.6%)
Race; *n (*%)	Asian	1 (4.5%)
	Black or African American	2 (9.1%)
	Mixed race	1 (4.5%)
	White	18 (81.8%)
Hispanic/Latino ethnicity; *n* (%)	Yes	5 (22.7%)
	No	17 (77.3%)
Education; *n (*%)	Some college	4 (18.2%)
	Two-year college degree	3 (13.6%)
	Four-year college degree	7 (31.8%)
	Some graduate/professional school	2 (9.1%)
	Graduate/professional school degree	6 (26.1%)
Body mass index (BMI); mean (SD)		29.7 (6.6)
Baseline tobacco use	Never	9 (40.9%)
	Former	8 (36.4%)
	Current	5 (22.7%)
Trauma history; *n (*%)	Combat	18 (78.3%)
	Military sexual trauma (MST)	8 (34.8%)
Baseline PTSD (CAPS-5); mean (SD)		41.0 (8.5)
30-Day post-intervention PTSD (CAPS-5); mean (SD)		22.1 (13.5)

**TABLE 2:** Inflammatory biomarker concentrations before and after MDMA-assisted group therapy.

Inflammatory biomarker (abbreviation; units)	Final N	T1: Mean (SD) concentration	T1: Mean (SD) concentration (natural log)	T1: Mean (SD) CV (%)	T2: Mean (SD) concentration	T2: Mean (SD) concentration (natural log)	T2: Mean (SD) CV (%)	Mean (SD) difference	Mean (SD) difference (natural log)	Hedges’ g	95% CI
Interleukin-6 (IL-6; pg/mL)	15	1.13 (0.81)	–0.13 (0.76)	12.42 (8.00)	1.16 (0.58)	0.014 (0.56)	11.56 (8.02)	0.03 (0.62)	0.14 (0.56)	0.24	–0.25, 0.72
Tumor necrosis factor alpha (TNF-α; pg/mL)	17	0.87 (0.30)	–0.19 (0.31)	5.80 (5.13)	0.84 (0.30)	–0.23 (0.31)	6.12 (4.01)	–0.03 (0.11)	–0.04 (0.14)	–0.24	–0.69, 0.23
C-reactive protein (CRP; ng/mL) ^†^	13	20.02 (17.06)	2.65 (0.86)	7.59 (3.46)	21.81 (15.30)	2.83 (0.76)	10.68 (7.16)	1.79 (15.79)	0.18 (0.73)	0.23	–0.30, 0.74
	10^‡^	13.00 (11.04)	2.32 (0.67)	7.33 (3.54)	18.53 (13.01)	2.69 (0.72)	9.40 (6.47)	5.56 (8.59)	0.37 (0.55)	0.61	–0.04, 1.22

† CV values associated with imputed concentrations were excluded from mean CV calculations. ^‡^Subset only includes participants for whom data was in the quantifiable range of the assay at both timepoints. Final N represents the number of participants with duplicate samples at both timepoints and CV ≤ 30%. For interpretability, descriptive statistics are reported as both raw data and natural log transformed values. Effect sizes and confidence intervals were calculated using natural log transformed data.

### 3.1 Changes in biomarkers

For IL-6, data for seven participants were excluded due to high (= 30%) CVs. Analysis of the remaining data (*n* = 15) yielded a small increase in IL-6 (Hedges’ *g* = 0.24; 95% CI = –0.25, 0.72) from baseline to end-of-intervention. For TNF-α, five participants had missing duplicates at one timepoint, and two of these also showed high CVs at the other timepoint. Findings for the remaining data (*n* = 17) demonstrated a small decrease in TNF-α (Hedges’ *g* = –0.24; 95% CI = –0.69, 0.23). For CRP, seven participants were excluded from analyses for concentration values exceeding the ULOQ at both timepoints. Two more participants were excluded due to high CVs, yielding a final sample size of *n* = 13, which showed a small increase in CRP (Hedges’ *g* = 0.23; 95% CI = –0.30, 0.74).

When including only participants having CRP concentrations within-range values at *both* timepoints (*n* = 10) the effect became substantially larger in magnitude (Hedges’ g = 0.61; 95% CI = –0.04, 1.22). Inspection of the data revealed that the large difference in effect between the two sample sizes was driven primarily by a single participant; removing this participant from analyses yielded a Hedges’ g effect size of –0.57 (95% CI = –1.12, 0.03).

When analyses were restricted to samples with a CV **≤** 20% at both timepoints (Supplementary Table 1), the effect size for IL-6 (*n* = 9) increased, becoming medium in size (Hedges’ *g* = 0.57; 95% CI = –0.10, 1.2). Restricting to CV ≤ 20% did not alter the TNF-α effect size, as sample size was unchanged. The effect size for CRP (*n* = 11) reflected primary findings (Hedges’ *g* = 0.28; 95% CI = –0.29, 0.83), which also applied when including participants having within-range values at *both* timepoints (*n* = 9; Hedges’ *g* = 0.64; 95% CI = –0.04, 1.30).

### 3.2 Correlations of biomarkers and PTSD symptom severity

Spearman’s rho correlations among variables of interest are shown in Table 3. Baseline IL-6 and TNF-α concentrations showed weak-to-moderate positive associations with baseline CAPS-5 scores (ρ = 0.45 and ρ = 0.32, respectively), where higher biomarker concentrations were associated with greater PTSD symptom severity. CRP demonstrated a negligible association with CAPS-5 scores. This association became negative and weak when including only participants for whom data was in the quantifiable range of the assay at both timepoints (ρ = –0.26), such that higher baseline CRP values were associated with lower PTSD symptom severity. End-of-intervention concentration values of IL-6, TNF-α, and CRP all yielded negligible associations with 30-day post-intervention CAPS-5 scores. When only using CRP concentrations for participants with data in the quantifiable range at both timepoints, the effect was negative and small (ρ = –0.27), where higher baseline CRP values were associated with lower PTSD symptom severity.

**TABLE 3:** Spearman’s rho correlations between inflammatory biomarkers and PTSD scores.

Inflammatory biomarker (abbreviation; units)	Final N	Baseline concentration vs. baseline CAPS-5 score	End-of-intervention concentration vs. 30-day post-intervention CAPS-5 score	Baseline concentration vs. change in CAPS-5 score	Change in concentration vs. change in CAPS-5 score
Interleukin-6 (IL-6; pg/mL)	15	0.45	0.10	–0.25	0.41
Tumor necrosis factor alpha (TNF-α; pg/mL)	17	0.32	–0.02	–0.14	0.05
C-reactive protein (CRP; ng/mL) ^†^	13	–0.07	–0.06	0.13	–0.26
	10^‡^	–0.26	–0.27	–0.38	–0.21

† CV values associated with imputed concentrations were excluded from mean CV calculations. ^‡^Subset only includes participants for whom data was in the quantifiable range of the assay at both timepoints. Final N represents the number of participants with duplicate samples at both timepoints and CV ≤ 30%. Change scores were calculated by subtracting baseline values from end-of-intervention values.

Higher baseline IL-6 concentrations were weakly associated with PTSD symptom improvement (ρ = –0.25), whereas associations between baseline TNF-α and CRP concentrations and change in CAPS-5 scores were negligible. For CRP, the relationship became negative, approaching a moderate magnitude when limited to participants with quantifiable data at both timepoints (ρ = –0.38), where higher baseline CRP was associated with greater PTSD symptom improvement.

Finally, a moderate association was found between the change in IL-6 concentration and change in CAPS-5 score (ρ = 0.41), in which increased IL-6 concentration over the intervention was associated with less PTSD symptom improvement. The association was negligible between the TNF-α and CAPS-5 change scores. For CRP, the relationship was weak and negative (ρ = –0.26), where increases in CRP over the intervention period were associated with PTSD symptom improvement. The finding was consistent when only using concentration values for participants with data in the quantifiable range at both timepoints (ρ = –0.21).

## 4 Discussion

In this exploratory study, we examined the concentrations of inflammatory biomarkers across the intervention period of a single-arm, open-label, pilot trial of a 12–16-week MDMA-assisted group therapy protocol for PTSD. Primary analyses yielded small increases in IL-6 and CRP, and a small decrease in TNF-α, from before to after MDMA-assisted group therapy for PTSD. Sensitivity analyses produced outcomes generally consistent with, and sometimes larger than, primary analyses. We also found several small-to-medium-sized associations of varied directionality among biomarker concentrations and PTSD symptoms. These included associations observed at baseline, as well as links between baseline biomarker levels and symptom change, and between changes in biomarkers and changes in symptoms. No findings reached statistical significance in this small exploratory pilot study; however, these effect sizes may contribute to future research designs with larger samples.

Contrary to our expectations, IL-6 and CRP demonstrated small increases over the intervention period, which further increased when stricter criteria were applied during sensitivity analyses. As PTSD-focused interventions generally involve exposure to and reprocessing of memories or reminders of traumatic events (Foa et al., 2019; Mithoefer et al., 2017; Resick et al., 2024; Sloan and Marx, 2019), they may confer a paradoxical effect on inflammation through repeated activation of the stress response over the course of treatment. The concurrent elevations we observed in IL-6 and CRP concentrations align with the well-known relationship in which IL-6 stimulates CRP synthesis, thus the two are often observed as increasing together (Del Giudice and Gangestad, 2018).

Though we anticipated decreases in IL-6 and CRP, our findings were similar to Zaccari et al. (2023), who tested a yoga intervention for PTSD and observed greater IL-6 increases with yoga relative to cognitive processing therapy, as well as CRP elevations in both groups 3 months post-intervention, all alongside PTSD symptom improvements. Yeh et al. (2023) also reported significantly increased IL-6 and CRP in response to both pharmacotherapy treatment with sertraline and interpersonal psychotherapy adapted to PTSD at 1 year follow-up, despite PTSD symptom improvements. In contrast, another study reported no overall differences in IL-6 and CRP before and after both mindfulness-based stress reduction and active control interventions for PTSD, though IL-6 and CRP increases were both significantly associated with decreased PTSD symptom severity (Shapira et al., 2022). We found diverging patterns between changes in IL-6 and CRP concentrations relative to PTSD symptom severity. Increases in IL-6 were moderately associated with smaller reductions in PTSD symptoms, whereas increases in CRP were weakly associated with greater PTSD symptom reduction. The mixed findings among our study and others may reflect differences in study design, sample characteristics, and intervention type, as well as the complex mechanisms regulating immune and stress responses.

Stronger effects for both IL-6 and CRP emerged in sensitivity analyses. For IL-6, the effect size increased when restricting analyses to samples with ≤ 20% CV values, indicating that the effect strengthened with greater assay precision. For CRP, the effect size increased when only including participants with concentrations in the quantifiable range at both timepoints (*n* = 10), as opposed to including those for whom the high standard was imputed at one of the two timepoints (*n* = 13). The difference in effect between the two sample sets appears to have been influenced disproportionately by a single participant whose baseline CRP value was imputed with the high standard. This individual showed the greatest reduction in CRP concentration across the entire sample, despite only a one-point change in CAPS-5 score. Excluding this individual from analyses (*n* = 12) produced an effect size consistent with the *n* = 10 sensitivity analysis. While the effect size increases for IL-6 and CRP are notable, interpretation is limited by small sample size.

Contrasted with the increases in IL-6 and CRP, and aligned with our expectations, we observed a small decrease in TNF-α concentration from before to after MDMA-assisted group therapy. This decrease was directionally consistent with an earlier preliminary analysis on a subset of 10 participants (Hedges’ *g* = –0.53; 95% CI = –1.16, 0.13), prior to completion of full assays. Interestingly, this finding differs from those of other PTSD intervention studies where TNF-α concentrations increased during psychotherapy (Himmerich et al., 2016; Toft et al., 2018) and 1 year after either pharmacotherapy or psychotherapy (Yeh et al., 2023). Although this contrast should be interpreted cautiously, it raises the possibility that MDMA-assisted therapy may exert distinct effects on TNF-α regulation, warranting further study.

Consistent with numerous cross-sectional studies (Yang et al., 2025), elevations in IL-6 and TNF-α were associated with greater PTSD symptom severity; however, these associations were only observed at baseline. End-of-intervention biomarker levels showed negligible associations with 30-day CAPS-5 scores, possibly attributable to a ∼32-day gap between end-of-intervention biomarker data collection and follow-up assessments. Conversely, there was an average of approximately 4 days between baseline biomarker data collection and baseline CAPS-5 assessment. Physiological recovery and immune re-regulation may occur more slowly than PTSD symptom reduction, and symptom improvements may have continued during the 32 days post-intervention as participants integrated therapeutic gains. Additionally, total CAPS-5 scores may have obscured nuanced associations, as biomarker changes may differ by symptom cluster (e.g., hyperarousal may align more closely with inflammation via HPA-axis reactivity). Although many studies report positive associations between CRP and PTSD symptoms (Yang et al., 2025), our findings of a negligible association between CRP and PTSD symptoms at baseline were consistent with those of Maples-Keller et al. (2022). Of note, a small negative correlation emerged following sensitivity analysis, with higher CRP concentrations weakly associated with lower PTSD symptoms. The relationship was maintained when examining the association between end-of-intervention CRP concentration and 30-day post-intervention PTSD symptoms.

We hope our findings will inform further research investigating changes in inflammatory biomarkers as potential physiological mechanisms of change for MDMA-assisted therapies. If these mixed findings in pro-inflammatory biomarker concentration changes are replicated across future studies, they may suggest complex modulation of inflammatory processes across treatment. Although we anticipated decreases in the pro-inflammatory biomarkers IL-6, TNF-α, and CRP across the intervention, it is possible that increases in IL-6 and CRP may correspond to trauma re-processing and integration, as well as a period of recalibration for nervous system regulation. To better understand the trajectory of inflammation in the context of MDMA-assisted therapy, future studies should measure inflammatory biomarker levels over an extended period, following treatment completion. Given the higher rates of inflammatory diseases observed in individuals with PTSD, it will also be important to examine inflammatory biomarker concentration trajectories in individuals with comorbid PTSD and inflammatory conditions (e.g., autoimmune disorders), as well as in those with conditions associated with chronic inflammation (e.g., substance use disorders).

Our novel exploratory study has several limitations. First, the study was not powered to detect significant effects, and biomarker research generally requires larger sample sizes (Janes et al., 2015); therefore, all findings should be interpreted with caution. Additionally, the limited sample size precluded the incorporation of factors associated with systemic inflammation—such as depressive symptoms (Mac Giollabhui et al., 2021), body mass index (BMI) (Khanna et al., 2022), smoking status (Gonçalves et al., 2011), and anti-inflammatory medication use (Hori and Kim, 2019)—into analytic models, limiting mechanistic inference. Furthermore, we were unable to use data from several participants for whom CV values exceeded 30%. Sample sizes were therefore smaller and varied across inflammatory biomarkers, further limiting statistical power. For CRP, concentration values for seven participants surpassed the upper limit of quantification at both timepoints. This is consistent with prior evidence that individuals with PTSD are more likely to exhibit clinically elevated CRP levels than those without the disorder (Spitzer et al., 2010). These participants were excluded from analyses, which limits interpretation, as individuals with values outside the quantifiable range may exhibit different patterns of change than those with in-range data at both timepoints. Exclusion of these data further limits statistical power, as well as the generalizability of findings to individuals with higher concentration values. Finally, as this was an exploratory sub-study that occurred within a single-arm pilot trial, there was no control group against which to compare outcomes. As such, findings cannot be attributed specifically to the MDMA-assisted therapy intervention.

## 5 Conclusion

To our knowledge, this is the first study examining changes in inflammatory biomarkers in the context of MDMA-assisted therapy. Taken together, our findings highlight the complexity of PTSD–inflammation relationships, which are shaped by substantial heterogeneity in both HPA-axis and immune modulation (Katrinli et al., 2022). PTSD symptoms themselves are also heterogeneous; for example, diagnostic subtypes have been associated with differential effects on neuroendocrine function (Jarkas et al., 2025). Further compounding complexity, intervention studies vary in design, modality, and sampling strategy, often yielding mixed results (Sumner et al., 2020). Additional research employing comparator groups and larger samples will be needed to determine whether these modest changes reflect random variation, measurement noise, or a true biological response to MDMA-assisted group therapy. A larger study sample would also permit more advanced modeling, including examination of biomarker–symptom cluster relationships while controlling for demographics and covariates such as depression symptoms (Mac Giollabhui et al., 2021), body mass index (Khanna et al., 2022), and use of anti-inflammatory medications (Rhein et al., 2024). These approaches could contribute to a clearer understanding of how inflammatory processes relate to PTSD symptoms and whether they are influenced by MDMA-assisted therapy.

## Data Availability

De-identified data may be made available upon reasonable request and with appropriate approvals in accordance with Veterans Administration policies.

## Footnotes

1
www.clinicaltrials.gov
