# Supplementary material for: Inflammatory biomarker outcomes associated with MDMA-assisted therapy: an open-label exploratory study
Source: Front Neurosci. 2026 Mar 23;20:1716817. doi: 10.3389/fnins.2026.1716817 (PMC13050828; doi:10.3389/fnins.2026.1716817)
Supplement: Supplementary file 1 [file Table_1.docx]

**Supplementary Table S1**. Post-hoc sensitivity analyses were conducted restricting the CV cutoff to ≤ 20%. For interpretability, descriptive statistics are reported as raw data and natural log transformed values. Effect sizes and confidence intervals were calculated using natural log transformed data.

| Inflammatory Biomarker  (abbreviation; units) | Final N | T1:  Mean (SD) Concentration | T1:  Mean (SD) Concentration  (natural log) | T1:  Mean (SD)  CV (%) | T2:  Mean (SD) Concentration | T2:  Mean (SD) Concentration  (natural log) | T2:  Mean (SD) CV (%) | Mean (SD) Difference | Mean (SD) Difference  (natural log) | Hedges’ g | 95% CI |
| --- | --- | --- | --- | --- | --- | --- | --- | --- | --- | --- | --- |
| Interleukin-6  (IL-6; pg/mL) | 9 | 0.93 (0.75) | -0.33 (0.77) | 10.10 (5.37) | 1.15 (0.65) | -0.03 (0.64) | 7.21 (4.34) | 0.22 (0.58) | 0.31 (0.49) | 0.57 | -0.10, 1.2 |
| Tumor necrosis factor alpha (TNF-α; pg/mL) | 17 | 0.87 (0.30) | -0.19 (0.31) | 5.80 (5.13) | 0.84 (0.30) | -0.23 (0.31) | 6.12 (4.01) | -0.03 (0.11) | -0.04 (0.14) | -0.24 | -0.69, 0.23 |
| C-reactive protein (CRP; ng/mL) | 11 | 17.15 (15.84) | 2.50 (0.83) | 6.99 (2.98) | 20.36 (15.89) | 2.74 (0.78) | 8.07 (4.18) | 3.20 (16.67) | 0.23 (0.78) | 0.28 | -0.29, 0.83 |
|  | 9^‡^ | 12.00 (11.26) | 2.24 (0.66) | 6.63 (2.93) | 18.18 (13.75) | 2.65 (0.75) | 7.82 (4.35) | 6.18 (8.88) | 0.41 (0.57) | 0.64 | -0.04, 1.30 |

^†^CV values associated with imputed concentrations were excluded from mean CV calculations.

^‡^Subset only includes participants for whom data was in the quantifiable range of the assay at both timepoints.
